# Charged alpha-helical peptides: unlocking a powerful new class of ion channel gating modifiers

**DOI:** 10.1007/s12551-026-01465-4

**Published:** 2026-09-22

**Authors:** Diego Santos Souza, Camila Dourado, Pedro V. Moraes-Santos, Jader Santos Cruz

**Affiliations:** 1 Department of Physiology, Federal University of Sergipe, São Cristóvão, Brazil; 2 Excitable Membranes Laboratory, Department of Biochemistry and Immunology, Federal University of Minas Gerais, Belo Horizonte, 31270-901 Brazil

**Keywords:** Ion channels, Gating modifiers, Amphipathic α-helical peptides, Membrane-mediated allostery, Voltage-gated sodium channels, Peptide toxins

## Abstract

Voltage-gated ion channels are commonly modulated by peptide toxins that alter channel gating through direct interactions with pore-forming or voltage-sensing domains. Most well-characterized gating modifiers belong to structurally constrained, disulfide-rich toxin families whose activity is largely explained by specific toxin-channel interactions. However, increasing evidence suggests that a diverse group of amphipathic α-helical peptides influences ion-channel function through mechanisms that extend beyond the classical ligand-receptor paradigm. Unlike canonical gating modifiers, these peptides frequently partition into the lipid bilayer, where membrane association, interfacial folding, and peptide-induced alterations in bilayer organization contribute to their biological activity. In this review, we examine the biophysical principles underlying peptide-mediated modulation of ion channels, with particular emphasis on voltage-dependent sodium channels. We discuss how changes in membrane surface charge, lipid packing, lateral pressure profiles, hydrophobic mismatch, and curvature stress may influence the energetic coupling between membrane proteins and their surrounding lipid environment, thereby affecting channel gating. Particular attention is given to poneratoxin and related aculeatoxins, which illustrate how relatively simple α-helical scaffolds can modulate voltage-gated sodium channels through mechanisms closely linked to membrane interactions. Collectively, these observations support a framework in which ion-channel modulation emerges from the coupled behavior of peptide, lipid bilayer, and membrane protein rather than from toxin binding alone. Understanding these interactions may provide new insights into membrane-mediated regulation of ion channels and inform future strategies for the development of modulators targeting the protein-lipid interface.

## Introduction

Virtually every physiological process, from the rapid computation of thoughts in the mammalian brain to the synchronized contraction of the heart, relies on the precise orchestration of transmembrane electrical signals. Far beyond merely propagating action potentials, these bioelectric fluxes serve as upstream regulators of intracellular metabolism, gene transcription networks, and protein trafficking (Hille 2001).

At the center of this scenario sits the ion channel protein superfamily: a diverse cohort of more than 140 structurally related pore-forming macromolecules (Yu and Catterall 2004). By acting as highly sensitive, voltage-gated nanomachines, these proteins maintain electrochemical gradients and drive complex signaling cascades across both excitable and non-excitable cells (Hille 2001). They share a common architecture consisting of a central pore domain, responsible for ion conduction, and four voltage-sensing domains (VSDs) that turn the channel on or off in response to changes in the transmembrane potential (Bosmans and Swartz 2010). These changes define the gating cycle of voltage-dependent ion channels (VDICs) and comprises three distinct states: closed (resting), open (activated), and in some cases, also an inactivated state.

Channel gating can be viewed as a dynamic redistribution among multiple conformational states, with changes in membrane voltage shifting the equilibrium toward specific structural configurations and thereby controlling channel activity.

Because of their central role in cellular regulation, voltage-dependent ion channels represent a premier frontline target in evolutionary warfare (Herzig et al. 2020). Over millions of years, predatory organisms have evolved an arsenal of potent peptide toxins designed to disrupt host physiology with lethal precision (Fry et al. 2009; Herzig et al. 2020).

Venom peptides modulate voltage-dependent ion channels through two principal mechanisms. One group act as pore blockers (PBs), binding within or near the ion-conducting pathway and physically obstructing ion permeation. Because the pore domain is largely solvent accessible, the affinity and selectivity of these toxins are primarily determined by direct peptide-protein interactions and are thought to be relatively insensitive to the surrounding lipid environment (Catterall et al. 2007; Aoki-Shioi et al. 2025). A second group, known as gating modifiers (GMs), targets the voltage-sensing domains (VSDs) and alters channel function by shifting the relative stability of resting, activated, or inactivated conformations (Bosmans and Swartz 2010), thereby modifying the kinetics and voltage dependence of gating.

Historically, studies of gating modifiers have focused primarily on structurally constrained, disulfide-rich peptide toxins, particularly those containing the inhibitor cystine-knot (ICK) motif found in arachnids, cone snails, and sea anemones (Catterall et al. 2007). Within this framework, modulation is generally attributed to direct toxin-channel interactions, whereby peptide binding alters the relative stability of channel conformations and thereby influences activation or inactivation processes (Cestèle and Catterall 2000; Sokolov et al. 2008; Wisedchaisri et al. 2021). More recently, attention has turned to a diverse group of amphipathic α-helical peptides whose effects on channel function appear to involve a more complex interplay between peptide structure, membrane organization, and channel conformational dynamics. These observations have prompted renewed interest in the possibility that membrane perturbation itself may contribute to gating modulation, either independently of or in conjunction with direct peptide-channel interactions.

Unlike pore blockers or classical voltage-sensor toxins, many amphipathic α-helical peptides exhibit a strong tendency to partition into the lipid bilayer. This mode of interaction places the peptide at the interface between the membrane and the channel, where it can influence the physicochemical environment surrounding membrane-embedded channel domains. Rather than relying exclusively on high-affinity recognition of discrete receptor sites, the effects of these peptides may arise from a combination of direct peptide-channel interactions and peptide-induced changes in bilayer organization. Such changes can include alterations in surface electrostatics (McLaughlin 1989), lateral pressure profiles, curvature stress, and bilayer thickness, all of which have been implicated in the regulation of membrane protein function. By modifying these parameters, amphipathic α-helical peptides have the potential to influence the energetic coupling between the lipid bilayer and voltage-sensing domains, thereby affecting channel activation, inactivation, and other gating-related conformational transitions. This perspective supports the view that channel modulation may emerge from the collective behavior of a peptide-lipid-protein system rather than from toxin binding alone.

This review provides a comprehensive biophysical synthesis of amphipathic α-helical peptides as an emerging class of unconventional ion-channel gating modifiers.

## NaV channels and gating modifiers

Gating modulation is observed in several voltage-dependent ion channel families, yet voltage-dependent Na^+^ (NaV) channels stand out as the leading model for exploring these allosteric interactions. By regulating membrane excitability, action potential initiation and propagation, and intracellular signaling pathways, these channels play critical roles in neuronal communication, muscle contraction, cell migration, and proliferation (Rhana et al. 2017, 2025).

Although NaV channels are best known for their roles in the central and peripheral nervous systems, skeletal muscle, and cardiac tissue, growing evidence indicates that they also contribute to the physiology and pathophysiology of non-excitable cells, including cancer cells (Rhana et al. 2017; Djamgoz 2024), making NaV channels important therapeutic targets in a broad spectrum of clinical disorders. From a structural and biophysical perspective, NaV channels provide a particularly informative model for investigating gating modulation because channel activation depends on the movement of positively charged S4 segments within the voltage-sensing domains (Catterall 2010; Jan 2025). These conformational transitions involve the displacement of gating charges through the membrane electric field and are inherently influenced by the physicochemical properties of the surrounding lipid environment (Kontis and Goldin 1997; Machtens et al. 2017). Consequently, factors such as membrane surface charge, lipid composition, and protein-lipid interactions can affect the relative stability of channel conformations and modulate transitions between resting, activated, and inactivated states (Duclohier and Pichon 2004) (Agwa et al. 2017, 2020; Qiao et al. 2024). Even subtle changes in the local membrane environment and/or electric field may therefore influence channel gating by altering the energetic coupling between voltage sensors and the lipid bilayer.

Unlike classical gating modifiers, which typically recognize well-defined receptor sites through highly structured and conformationally constrained molecular scaffolds (Dyson 2011), many α-helical peptides appear to derive their activity from a more complex interplay between peptide structure, membrane organization, and channel function. In these systems, membrane association is not simply a prerequisite for target access but may contribute directly to the modulation process itself (Deplazes 2017).

It is known that amphipathic α-helical peptides readily partition into lipid bilayers due to the spatial segregation of hydrophobic and charged residues along opposite faces of the helix. Nevertheless, others suggest that this structural arrangement is not strictly required for membrane interaction. Upon approaching the membrane interface, these peptides frequently undergo disorder-to-helix transitions that are thermodynamically driven by favorable hydrophobic partitioning and electrostatic interactions with lipid headgroups (Lomize et al. 2007; Corin and Bowie 2022). This membrane-induced folding process increases peptide residence time within the interfacial region of the bilayer and promotes intimate coupling between peptide structure and membrane organization (Lomize et al. 2007; Corin and Bowie 2022).

Such perturbations are increasingly recognized as important determinants of membrane protein function. Contemporary models of membrane-mediated allostery propose that changes in bilayer physical properties can influence embedded proteins by altering the energetic cost of conformational transitions (Rusinova et al. 2021). In voltage-gated ion channels, modifications in membrane thickness, elasticity, surface charge, or lateral pressure profiles may affect voltage-sensor movement, pore opening, inactivation kinetics, and the coupling between voltage-sensing and pore domains (Schow et al. 2012). Within this framework, the lipid bilayer contributes to channel regulation by providing a physical environment that influences the stability and interconversion of functional states.

**Fig. 1:**
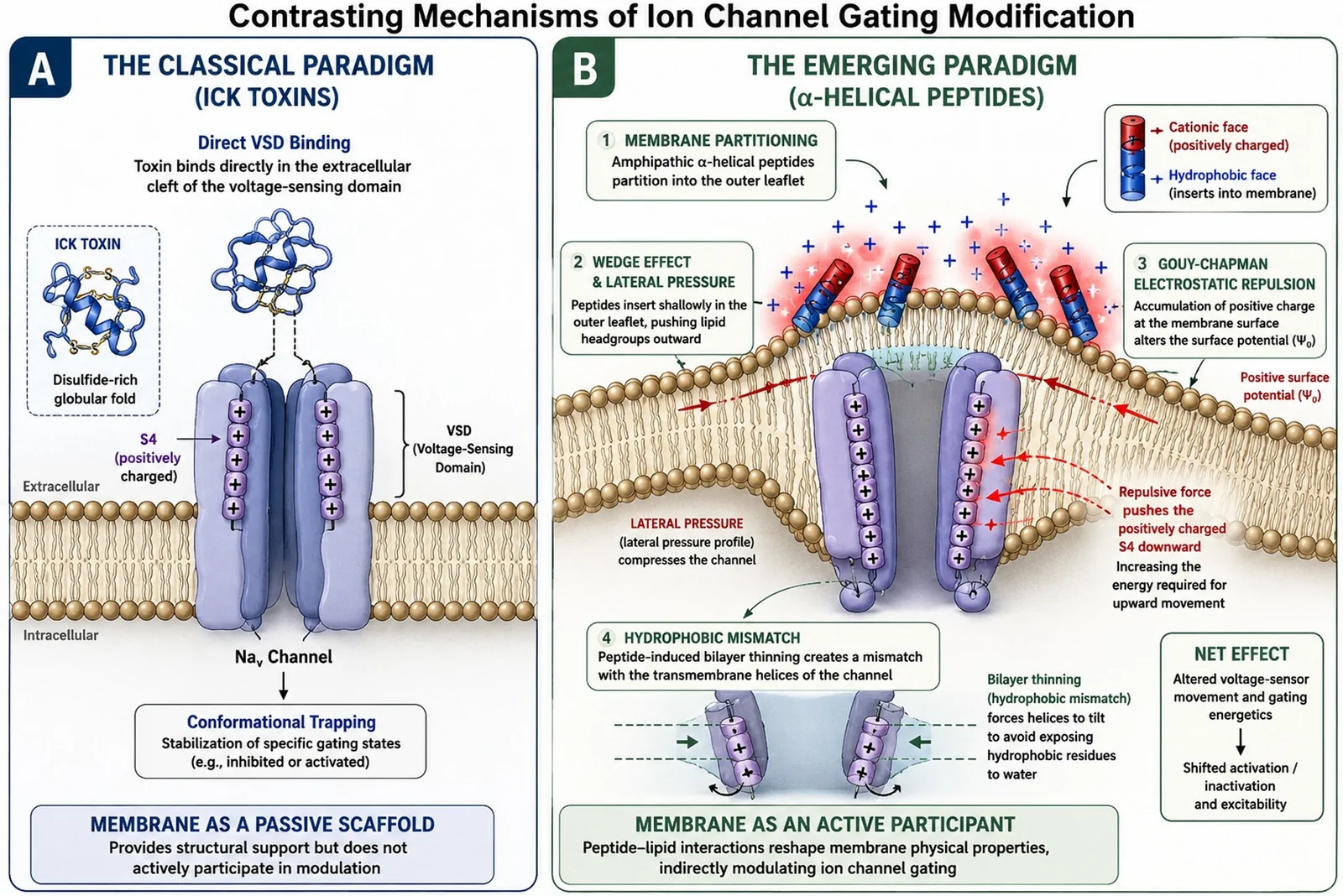
Contrasting mechanisms of ion-channel gating modification. Classical disulfide-rich gating modifiers stabilize specific channel conformations through direct interactions with voltage-sensing domains, whereas amphipathic α-helical peptides modulate channel gating through membrane-mediated allosteric mechanisms

Taken together, these observations suggest that neutral and charged α-helical peptides can influence ion-channel function through mechanisms that extend beyond conventional ligand-receptor interactions (Fig. 1). By modifying bilayer properties such as surface charge, lipid packing, membrane curvature, and lateral pressure profiles, these peptides have the potential to alter the coupling between membrane proteins and their surrounding lipid environment. The resulting effects on channel conformational equilibria provide a mechanistic framework for understanding how membrane-associated peptides can modulate gating without relying exclusively on direct interactions with canonical receptor sites. This perspective broadens the traditional view of gating modulation and highlights the importance of considering the peptide-lipid-protein system as an integrated functional unit.

## Allosteric modulation of ion channels by amphipathic α-helical peptides: structural conditionality and non-canonical gating modulation

The classical understanding of ion-channel gating modulation has been largely grounded in the ligand-receptor paradigm, whereby structurally rigid, disulfide-rich toxins interact with well-defined protein epitopes to alter channel function. However, biophysical evidence has established neutral or charged amphipathic α-helical peptides as a mechanistically distinct class of modulators (Avci et al. 2018). The defining characteristic of these molecules is not merely their intrinsic helical conformation or lipid affinity, but rather the sophisticated manner in which these properties are coupled to channel function through the membrane interface. Instead of relying on a single protein–ligand interaction surface, the pharmacology of these peptides emerges from the thermodynamically coupled behavior of a tripartite system comprising peptide, lipids, and ion channels (Agwa et al. 2017; Xu et al. 2019; Saponaro and Lolicato 2022).

A central feature underlying the biological activity of these peptides is the conditional nature of their structure. In aqueous solution, many of these molecules exist as intrinsically disordered or only partially structured polypeptide chains, acquiring a stable α-helical conformation only upon encountering a lipid environment (Narayanaswami et al. 2010). This membrane-induced folding transition is not a secondary structural event but rather a component of the modulation mechanism itself (Halskau et al. 2008). Interfacial association with the membrane reduces conformational degrees of freedom and promotes the amphipathic organization of amino-acid side chains. In this configuration, cationic residues such as arginine and lysine remain concentrated near the membrane-water interface, where they can modify local electrostatic conditions and potentially influence the environment experienced by nearby voltage-sensing domains.

From a thermodynamic perspective, the favorable enthalpy associated with lipid partitioning and intramolecular hydrogen-bond formation can compensate for the entropic cost of peptide folding. Consequently, membrane association stabilizes the α-helical conformation while promoting sustained interactions with the bilayer interface. The resulting peptide-membrane complex provides a physical basis through which changes in bilayer organization and local electrostatics may be coupled to ion-channel function.

## Pharmacological paradigms: poneratoxin and aculeatoxins

The conceptual framework of membrane-mediated modulation is exemplified by natural toxins derived from ant venoms, which have evolved to exploit these physicochemical principles with remarkable specificity toward voltage-gated sodium (NaV) channels (Thapa et al. 2024).

### Poneratoxin (PoTX) and gating remodeling

Poneratoxin (PoTX), a 25-residue neuropeptide isolated from the venom of the bullet ant *Paraponera clavata*, represents the archetype of this molecular strategy. Biophysical studies employing dodecylphosphocholine (DPC) micelles as membrane-mimetic systems revealed that PoTX adopts a bent “V-shaped” architecture characterized by a helix-turn-helix motif, with the hinge centered around residues Phe12 and Gly13. The absence of a rigid globular fold supports the notion that the toxin has been evolutionarily optimized for dynamic interfacial partitioning rather than for classical lock-and-key binding to a discrete extracellular receptor (Szolajska et al. 2004; Lü et al. 2025).

Electrophysiological studies have demonstrated that PoTX delays NaV-channel inactivation, generating a persistent sodium current and shifting channel activation toward membrane potentials approximately 40 mV more negative than those observed under control conditions (Szolajska et al. 2004; Lü et al. 2025).

### Aculeatoxins: variations on a conserved molecular framework

The mechanistic principles exemplified by PoTX are further reinforced by the evolutionary clade of aculeatoxins, a family of vertebrate-selective and highly algogenic venom peptides that includes Ta3a, Mri1a, and Rm4a (Robinson et al. 2023). Among these, Rm4a, isolated from *Rhytidoponera metallica*, provides an elegant example of this class. Rm4a is a 26-residue peptide that is notably devoid of cysteine residues and therefore lacks the disulfide bridges that characterize most classical gating modifiers (Robinson et al. 2023). The functional integrity of Rm4a is consequently not dependent on covalent structural constraints but instead arises from a physicochemical design enriched in positively charged residues and hydrophobic domains that promote the formation of a stable amphipathic α-helix upon membrane association (Robinson et al. 2023; Isaksen et al. 2025). A comparative analysis is presented in Table 1.

**Table 1:** Representative ant venom-derived α-helical peptides that modulate voltage-gated sodium channels. The table summarizes selected members of the aculeatoxin family and related peptides, highlighting their biological origin, structural characteristics, and functional effects on NaV channel activity

Peptide	Biological Source	Structural Features/Molecular Mass	Primary Target and Mechanism
PoTX	*Paraponera clavata*	25 residues; helix-turn-helix motif; ~ 2.76 kDa	Fast and slow NaV channels; inhibits voltage-dependent inactivation
Rm4a	*Rhytidoponera metallica*	26 residues; cysteine-free; amidated C-terminus	Mammalian NaV1.6 and NaV1.7 channels; induces nociceptive currents
Ta3a	*Tetramorium africanum*	Cationic, amphipathic, membrane-induced α-helix	Vertebrate-selective; promotes robust cellular depolarization

Importantly, the carboxyl terminus of Rm4a is naturally amidated, a post-translational modification that neutralizes the terminal carboxylate group and enhances the overall positive charge of the molecule while increasing resistance to proteolytic degradation. Such features are likely to improve membrane affinity and electrostatic interactions at the membrane interface. Injection of aculeatoxins produces robust nocifensive behaviors through direct hyperactivation of NaV isoforms, particularly NaV1.7, a channel critically involved in pain signaling (Robinson et al. 2023; Cummins 2025). The remarkable efficacy of this toxin family is highly informative because it demonstrates that profound alterations in channel gating can emerge from relatively subtle sequence variations embedded within a conserved physicochemical framework. Rather than relying on structurally complex receptor-recognition motifs, these peptides appear to exploit charge distribution, amphipathicity, and membrane responsiveness to influence ion-channel conformational dynamics.

## Lipid-mediated allostery and gating dynamics

The lipid bilayer is not merely a passive structural support for membrane proteins but an active allosteric participant in ion-channel biology. Bilayer thickness, transmembrane topology, membrane elasticity, and surface charge collectively shape the free-energy landscape that governs channel conformational transitions. Consequently, any molecule capable of modifying these parameters has the potential to influence channel function indirectly through membrane-mediated mechanisms (Patrick et al. 2018; Ponce 2026; Idikuda et al. 2026).

Host-defense peptides and antimicrobial peptides (AMPs) provide compelling conceptual support for this framework. Although traditionally associated with membrane permeabilization and pathogen lysis, many AMPs exert biological activity at sub-lytic concentrations by altering membrane architecture and mechanics. These peptides often display highly cationic amphipathic helices, with net charges ranging from + 7 to + 9, values remarkably similar to those observed in many venom-derived α-helical toxins. The mechanisms through which these molecules interact with membranes therefore provide valuable insight into how venom peptides may regulate ion channels through analogous physicochemical pathways (Idikuda et al. 2026).

## Hydrophobic mismatch

Hydrophobic mismatch represents one of the most thoroughly characterized physical mechanisms through which membrane-active peptides may influence ion-channel behavior. This phenomenon arises when the hydrophobic thickness of the lipid bilayer differs from the hydrophobic span of an embedded membrane protein. Such incompatibility is energetically unfavorable because it risks exposing hydrophobic surfaces of either the protein or the lipid core to the aqueous environment (Lundbæk 2008).

To minimize this energetic penalty, the membrane deforms locally through thinning, thickening, compression, or bending. Because ion-channel conformational states are coupled to their membrane environment, these deformations can directly alter channel energetics and shift the equilibrium between open, closed, and inactivated states.

Amphipathic α-helical peptides typically insert parallel to the membrane surface at the hydrophilic-hydrophobic interface rather than spanning the bilayer completely. Acting as molecular wedges, they induce localized expansion or compression of lipid packing. Channels mechanically coupled to this altered microenvironment experience a corresponding change in the energetic balance between functional states (Lundbæk 2008; Ponce 2026; Idikuda et al. 2026).

The conformational state that minimizes the total free energy of the integrated peptide-lipid-protein system will therefore be preferentially stabilized. This framework provides a compelling explanation for why a single class of amphipathic peptides can modulate a wide range of ion-channel families, including NaV, CaV, KV, TRP, and ASIC channels, without requiring the evolution of highly specialized receptor-binding interfaces.

## Electrostatic perturbation and voltage-sensor displacement

Biological membranes possess highly organized electrostatic environments that play a fundamental role in ion-channel function. According to Gouy-Chapman-Stern theory, membrane surface potential is strongly influenced by the distribution of charged lipids and ions adjacent to the phospholipid headgroup region. Consequently, molecules that accumulate at the membrane interface can significantly modify local electrostatic fields.

When cationic aculeatoxins or PoTX insert into the outer leaflet of the membrane, their exposed lysine- and arginine-rich surfaces increase the local positive charge density. This redistribution of charge alters the electrical double layer and modifies the electrostatic environment experienced by nearby voltage-gated channels (Lundbæk 2008; Robinson et al. 2023; Ponce 2026; Idikuda et al. 2026).

**Fig. 2:**
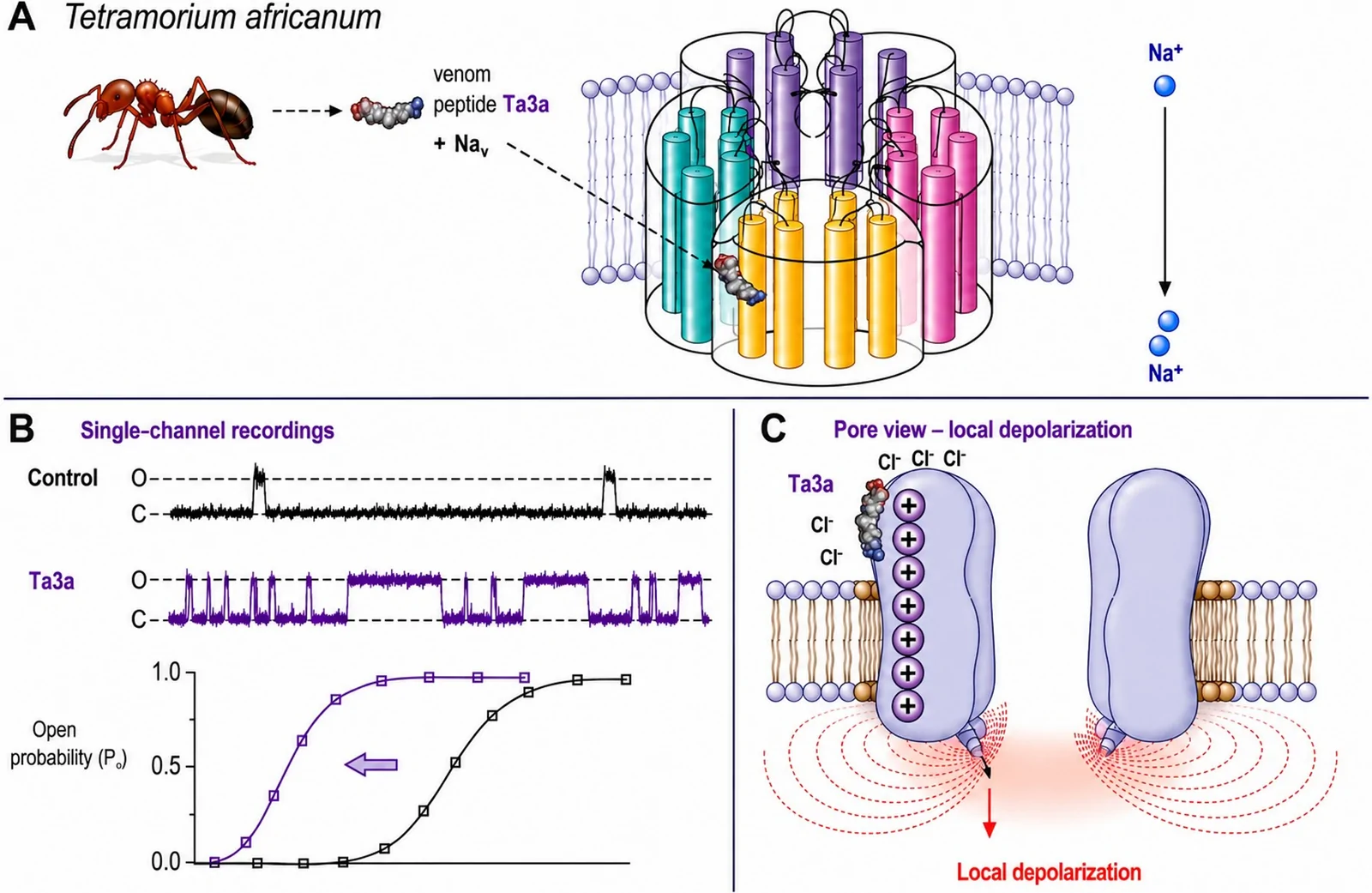
Proposed mechanism of NaV-channel modulation by the aculeatoxin Ta3a. **A** Schematic representation of the selective ant venom peptide Ta3a from *Tetramorium africanum* and its interaction with NaV channels. Unlike classical gating modifiers that bind to well-defined extracellular receptor sites, Ta3a is proposed to modulate channel activity through membrane-associated interactions and local electrostatic effects. **B** Representative single-channel recordings showing increased channel activity in the presence of Ta3a, accompanied by a leftward shift in the open probability (Po)-voltage relationship, indicating enhanced channel activation at more hyperpolarized membrane potentials. **C** Proposed mechanism underlying channel modulation. By changing the extracellular membrane surface potential Ta3a reduces the threshold for activation of NaV. This effect can be predicted by the Gouy-Chapman-Stern charge screening model. For more details, refer to Thapa et al. (2024). Adapted from Thapa et al. (2024)

Activation of voltage-gated channels is driven by the movement of conserved positively charged residues located within the S4 transmembrane segments of voltage-sensing domains. Because this movement occurs through a strong electric field, even modest changes in local electrostatics can substantially influence gating energetics. By accumulating positive charge at the membrane surface, amphipathic α-helical peptides may alter the free-energy cost associated with voltage-sensor displacement. The resulting changes in surface potential effectively shift channel activation and inactivation curves toward more hyperpolarized membrane potentials (Fig. 2). Consequently, channel modulation can occur without the need for stable toxin binding to extracellular channel loops. In such cases, electrostatic accumulation mediated by membrane partitioning becomes the pharmacological mechanism itself (Robinson et al. 2023; Ponce 2026).

## Curvature stress and mechanosensitive gating

An important consequence of the asymmetric insertion of amphipathic peptides into a single membrane leaflet is the generation of spontaneous membrane curvature and alterations in lateral pressure profiles. Because biological membranes are mechanically heterogeneous structures, perturbations introduced at the lipid interface can propagate over nanometer scales and influence the behavior of neighboring membrane proteins.

The insertion depth of an amphipathic α-helix determines how membrane lipids reorganize around the peptide. Shallow insertion tends to separate phospholipid headgroups, whereas deeper penetration can compress acyl chains and modify local membrane thickness (Molugu et al. 2022). These structural rearrangements generate curvature stress and redistribute mechanical forces throughout the bilayer (Robinson et al. 2023; Ponce 2026).

The concept of lateral pressure profiles provides a particularly useful framework for understanding these effects. Distinct regions of the membrane experience different mechanical stresses arising from lipid packing constraints, hydration forces, and hydrophobic interactions. Because ion-channel gating often involves large-scale movements of transmembrane helices and changes in protein cross-sectional area, the energetic cost of these conformational transitions is directly influenced by the surrounding pressure landscape (Samuli Ollila et al. 2011; Bavi et al. 2016).

Mechanosensitive ion channels are especially responsive to such perturbations. According to the “force-from-lipids” principle, mechanical forces generated within the bilayer are transmitted directly to the channel protein, thereby driving conformational transitions. Under this model, membrane tension itself becomes the gating stimulus (Yoshimura and Sokabe 2010; Bavi et al. 2016).

Consequently, peptides capable of modifying membrane curvature, elasticity, thickness, or tension possess the potential to regulate mechanosensitive channels without directly contacting the protein. By increasing local membrane stress, these molecules may favor conformational states that relieve elastic strain, thereby altering channel open probability and gating kinetics.

Although the majority of current evidence derives from studies of voltage-gated sodium channels, the same physical principles are likely applicable to numerous channel families, including PIEZO channels, TREK potassium channels, TRP channels, and acid-sensing ion channels (ASICs). This observation considerably broadens the physiological relevance of amphipathic α-helical toxins and suggests that membrane mechanics may represent a universal pathway for peptide-mediated channel regulation (Cox et al. 2019; Ruan et al. 2021).

Importantly, curvature stress, hydrophobic mismatch, and electrostatic remodeling should not be viewed as independent mechanisms. Rather, they represent interconnected manifestations of membrane perturbation. A single peptide may simultaneously alter bilayer thickness, modify surface potential, redistribute lateral pressure, and induce local curvature. The biological response therefore emerges from the integrated effect of multiple membrane-dependent processes acting on a shared energetic landscape.

## Conclusions and future perspectives

The examples discussed in this review reinforce the growing recognition that ion-channel modulation cannot always be explained solely through direct toxin-receptor interactions. In many systems, peptide activity reflects a coordinated interplay among membrane association, bilayer organization, and channel conformational dynamics. This concept is especially relevant for amphipathic α-helical peptides, whose actions arise from the combined behavior of peptide, lipid bilayer, and membrane protein instead of the occupation of a single high-affinity site.

Studies of poneratoxin and related aculeatoxins illustrate this principle clearly. Although these peptides lack the rigid, disulfide-rich architectures typically associated with classical gating modifiers, they exert pronounced effects on NaV-channel function through mechanisms closely linked to membrane partitioning and interfacial interactions. Their behavior emphasizes the need to view the lipid bilayer as an active determinant of channel regulation, not simply as a structural scaffold in which gating occurs.

From a biophysical perspective, hydrophobic mismatch, electrostatic remodeling, curvature stress, and changes in lateral pressure profiles provide complementary frameworks for understanding how membrane-associated peptides influence ion channels behavior. These processes likely act in combination, shaping the energetic coupling between membrane proteins and their surrounding lipid environment.

Several important questions remain open that should be addressed in near future: (1) Can membrane remodeling itself be considered a bona fide mechanism of gating modulation, or must modulation always involve direct stabilization of specific channel conformations? (2) To what extent are the effects of amphipathic α-helical peptides determined by direct peptide-channel interactions versus peptide-induced alterations in bilayer organization? (3) Is there a threshold of membrane perturbation required to influence channel gating, or can subtle local changes in electrostatics, curvature, or lipid packing produce measurable functional effects? (4) Could some toxins currently classified as conventional gating modifiers derive part of their activity from bilayer-mediated effects that have remained experimentally unresolved? (5) Does the lipid bilayer act merely as a medium through which channel modulation occurs, or should it be considered an integral component of the gating machinery itself? (6) Can peptide-induced membrane perturbations be visualized directly and correlated quantitatively with voltage-sensor movement, channel-state occupancy, and gating energetics?

Progress will require integrative approaches that combine electrophysiology, molecular dynamics simulations, quantitative membrane biophysics, cryogenic electron microscopy, high-resolution NMR spectroscopy, and emerging structural proteomic methods.

A deeper understanding of peptide-lipid-protein interactions may also have significant pharmacological implications. Future modulators could act at the protein-lipid interface as a means of regulating channel function, expanding current strategies in ion channel pharmacology and creating new opportunities for therapeutic development in disorders of excitability.

Amphipathic α-helical peptides therefore provide a valuable working model for examining how membrane organization shapes ion channel behavior. Continued investigation of these systems will refine our understanding of the relationship between membrane biophysics and channel gating and broaden the conceptual domain of ion-channel modulation.

## Data Availability

No datasets were generated or analysed during the current study.
